# Verbal Mediation During Auditory Equivalence Class Formation Using Go/No-Go Successive Matching-to-Sample

**DOI:** 10.1007/s40616-024-00209-3

**Published:** 2024-09-30

**Authors:** Charles S. Dingus, Robbie J. Hanson, Caio F. Miguel, Sydney Stern, Denys Brand

**Affiliations:** 1 Department of Psychology, California State University, Sacramento, 6000 J Street, Sacramento, CA 95819 USA; 2 College of Education and Human Services, Lindenwood University, St. Charles, MO USA

**Keywords:** Equivalence, Matching-to-sample, Protocol analysis, Successive matching, Verbal mediation

## Abstract

Successive matching-to-sample (S-MTS) with a go/no-go response requirement has previously produced equivalence classes with nonverbal auditory stimuli among college students. When participants are required to talk aloud during posttests (protocol analysis), their verbal behavior tends to match their selection performance. However, in some cases, the protocol analysis seems to interfere with posttests, in that equivalence yields are lower when participants are required to talk aloud. Thus, the current study replicated and extended previous research by requiring participants to complete emergence posttests before introducing training for the protocol analysis. Subsequently, participants completed one additional block of the transitivity/equivalence posttest with the talk-aloud requirement. Additionally, participants completed tact and intraverbal tests following emergence posttests to further assess possible verbal-mediation strategies. The results showed that six of eight college students formed equivalence classes, suggesting that previous failures could have been influenced by the talk-aloud requirement. Further, there was a positive correlation between verbal and nonverbal (selection) responses suggesting the possibility that verbal mediation may have contributed to equivalence-class formation.

Matching-to-sample (MTS) is commonly used for teaching conditional discriminations (e.g., if A1, then B1; if A2, then B2) in laboratory (Cumming & Berryman, 1965) and clinical settings (Shawler et al., 2023). Typically, MTS includes the presentation of a sample stimulus, an observing response, an array of comparisons, a learner’s selection of the comparison that corresponds to the sample, reinforcement (during training), and an intertrial interval (Sidman & Tailby, 1982). One of the important outcomes of MTS is that samples and comparisons may become mutually substitutable or equivalent (Sidman, 2000).

Despite its wide use, several alternatives to traditional MTS have been developed (e.g., Canovas et al., 2015; Debert et al., 2007; Lantaya et al., 2018). One such alternative is the go/no-go successive matching-to-sample (S-MTS; Frank & Wasserman, 2005) during which selecting the sample causes it to disappear and a comparison to appear in its place. Participants are taught to select (i.e., go) the comparison (i.e., S^+^) when it belongs to the same class as the sample, and to refrain (i.e., no go) from selecting the comparison (i.e., S^−^) when it does not (e.g., Howland et al. 2021; Lantaya et al., 2018; Zhelezoglo et al., 2021). Given that samples and comparisons appear successively (i.e., one at a time) in the same location, the S-MTS arrangement may prevent the development of location/position bias (Da Hora et al., 2019), eliminate the requirement for simultaneous simple discriminations (Green, 2001), and allow for the establishment of purely auditory classes (Hanson & Miguel, 2021; Hanson et al., 2022; Sordello et al., 2024).

Hanson and Miguel (2021) and Hanson et al. (2022) utilized S-MTS to teach conditional relations with verbal and nonverbal (i.e., common sounds) auditory stimuli. Their results showed that, in addition to establishing equivalence classes among auditory stimuli, adult participants could also vocalize stimulus names in a class-consistent manner. For instance, participants would say, “Boat [A1] goes with Pear [B1] and Flea [C1]” or “Rocket [A2] goes with Razor [B2] and Film rewinding [C2]^1^”. The authors suggested that sample and comparisons’ names may have become intraverbally related when, during S-MTS training, participants repeated the verbal auditory stimuli (i.e., echoed) or labeled nonverbal auditory stimuli (i.e., tacted) prior to receiving reinforcement for their selection responses. Even though no overt vocalizations were observed, it is possible that during equivalence tests, participants continued to engage in these intraverbal chains, the product of which may have generated supplemental stimulation to guide their test performance (e.g., Chastain et al., 2022; Jennings & Miguel, 2017; see Miguel, 2018 for a conceptual analysis).

To further investigate whether participants were engaging in verbal mediation during testing, Sordello et al. (2024) used a protocol analysis in which eight adult participants who underwent S-MTS training with nonverbal (everyday sounds) auditory stimuli were instructed to talk aloud during emergence posttests. The results showed that participants who met emergence criterion correctly tacted and engaged in class-consistent intraverbals while completing S-MTS tasks. However, only four of eight participants met emergence criterion, so it was unclear whether the requirement to talk aloud during emergence posttests interfered with participants’ performance during S-MTS tasks. Thus, the current study replicated and extended the results of Sordello et al. by requiring participants to first complete transitivity/equivalence posttests without talking aloud, and subsequently repeating the posttests with the talk aloud requirement. Moreover, participants completed tact and intraverbal tests following emergence posttests to assess possible verbal-mediation strategies.

## Method

### Participants, Setting, and Materials

Participants were eight typically developing adults, aged 19–44 years (*M* = 24), enrolled in undergraduate psychology courses who received extra credit for their participation. All sessions were conducted in a campus laboratory. Each participant sat at a table in front of a touchscreen computer while the primary investigator and a secondary observer sat approximately 0.5 m behind and next to the participant, respectively. Participants were presented with stimuli via Microsoft’s Visual Basic^®^ on the touchscreen computer. Pretraining and experimental stimuli were identical to Sordello et al. (2024; see Table 1). Participants received written instructions for each condition (Hanson et al., 2022; see Table 2). The university’s institutional review board approved all procedures and recruitment protocols.

**Table 1:** Pretraining and experimental stimuli

Phase	Class	A	B	C
Pretraining	1	Spoon	Fork	Knife
	2	Blue	Green	Red
Experimental	1	(ice in a cup)	(chopping vegetables)	(sliding door)
	2	(toaster popping)	(scanner)	(pen writing)
	3	(rocket engine)	(electric razor)	(film rewind)

*Note*. Pretraining stimuli were presented as dictated words from the computer's speaker and experimental stimuli were presented as an audio clip of that sound from the computer's speaker

**Table 2:** Written instructions for each condition

Condition	Instruction
Symmetry & transitivity/equivalence pretest	Once the task begins, you will hear a sound from the computer. After you hear the sound, a green box will appear on the screen. Touch the green box to hear a second sound. After you hear the second sound, a white box will appear on the screen. If you think the first and second sounds go together, touch the white box and say “click.” If the sounds do not go together, then do not touch the box and wait for the box to disappear. During this phase, you get no points or sounds as feedback. Remember, you can read these instructions at any time. Place the instructions in the designated location.
Baseline training	During this phase you will learn how to group sounds together. Once the task begins, you will hear a sound from the computer. After you hear the sound, a green box will appear on the screen. Touch the green box to hear a second sound. After you hear the second sound, a white box will appear on the screen. If you think the first and second sounds go together, touch the white box and say “click.” If the sounds do not go together, then do not touch the box and wait for the box to disappear. You will get points when sounds go together, and you will not get points when they do not go together. For the first few trials I will help you with the answer. After that you will have 4 seconds to respond on your own. If you do not respond, I will help you. You can read these instructions at any time. Place the instructions in the designated location.
Baseline test	Continue touching the white box and saying “click” for sounds that go together as before. During this time no points or sounds will be presented. Remember, you can read these instructions at any time. Place the instructions in the designated location.
Symmetry & transitivity/equivalence posttest	This is a new phase. Use what you have learned so far to figure out what sounds go together. Once the task begins, you will hear a sound from the computer. After you hear the sound, a green box will appear on the screen. Touch the green box to hear a second sound. After you hear the second sound, a white box will appear on the screen. If you think the first and second sounds go together, touch the white box and say “click.” If the sounds do not go together, then do not touch the box and wait for the box to disappear. During this phase, you will get no points or sounds as feedback. Remember, you can read these instructions at any time. Place the instructions in the designated location.
Condition	Instruction
Protocol analysis	We are interested in how people solve problems. We want you to think out loud for the duration of the experiment. So that you understand what we mean by thinking out loud, an example would be solving a math problem while vocally describing each step that you take while solving it. For example, if I were to solve the problem 2 x 20, I would say aloud, “First I would put the 2 under the 0. Then I would multiply 0 by the 2, making 0, and I would write the 0 under the problem. Next, I would multiply the bottom 2 by the top 2, getting 4. Finally, I would write the 4 under the problem, next to the 0, making a total of 40.” Try this now with problem number 1 (below). We are not concerned with whether you get the problem correct or how quickly you solve it. You will continue to speak aloud for the remainder of the study. Problem 1: 12 x 5 Problem 2: 11 x 4 Problem 3: 10 x 6 Problem 4: 5 x 10
Tact test	Once the task begins, you will hear a sound from the computer. You will have 5 seconds to tell me what the sound is for each sound you hear. I will not provide feedback. Remember, you can read these instructions at any time. Place the instructions in the designated location. Continue to speak aloud as you complete the tasks.
Intraverbal test	Once the task begins, you will hear a sound from the computer. You will have 5 seconds to respond with two sounds that go with the sound that you heard. I will not provide feedback. Remember, you can read these instructions at any time. Place the instructions in the designated location. Continue to speak aloud as you complete the tasks.

*Note.* Participants were presented instructions on separate sheets of paper and were required to read instructions silently and then summarize them aloud to the primary investigator prior to each condition

### Dependent Variables

The primary dependent variable was the percentage of correct go and no-go trials across conditions, defined as correct and unprompted responses (i.e., touching related sample and comparison combinations and refraining from touching unrelated combinations; Lantaya et al., 2018). Secondary dependent variables included the percentage of unique and experimenter-defined tacts during tact tests and the frequency of consistent, inconsistent, and irrelevant vocal-verbal statements (Chastain et al., 2022; Sordello et al., 2024) during the protocol analysis and intraverbal test (see Table 3 for secondary dependent variable definitions). Additionally, data for trials to criterion during baseline training and reaction times across conditions were collected. Reaction time was recorded for go-trials following the presentation of the white box and either the participant’s response or the end of the trial (i.e., refraining from touching the white box resulted in a reaction time of 8 s).

**Table 3:** Secondary dependent variable definitions

Secondary dependent variables	Definition
Experimenter-defined tacts	A vocal response that was similar to the tact that was determined by the experimenter (e.g., participant provided the tact “ice falling into a cup” when presented the sound defined by experimenters as “ice falling into a glass”).
Unique tacts	A vocal response that was dissimilar to the tact determined by the experimenter (e.g., participant provided the tact “the sound of a DJ scratching a record” when presented the sound defined by experimenters as “pen writing a signature”).
Consistent statements	Any vocal-verbal statement(s) that correctly identified two or more auditory stimuli as being related or unrelated (e.g., “These go together” when presented “chopping vegetables” [B1] as the sample and “sliding door” [C1] as the comparison, or responds, “These do not go together” when presented “signature” [C2] as the sample and “razor” [B3] as the comparison, or during the intraverbal test, the participant responds “rewind” [C3] and “razor” [B3] when presented the auditory stimulus for “rocket” [A3]).
Inconsistent statements	Any vocal-verbal statement(s) that incorrectly identified two or more auditory stimuli as being related or unrelated (e.g., “These go together” when presented “sliding door” [C1] as the sample and “scanner” [B2] as the comparison, or responded, “These do not go together” when presented “rewind” [C3] as the sample and “razor” [B3] as the comparison, or during the intraverbal test, the participant responds “rocket” [A3] and “sliding door” [C1] when presented the auditory stimulus for “scanner” [B2]).
Irrelevant statements	Any vocal-verbal statement that did not meet the aforementioned operational definitions (e.g., “I’m trying to figure this one out,” when presented the auditory stimulus for “chopping vegetables” [B1]).

### Procedure

**Fig. 1:**
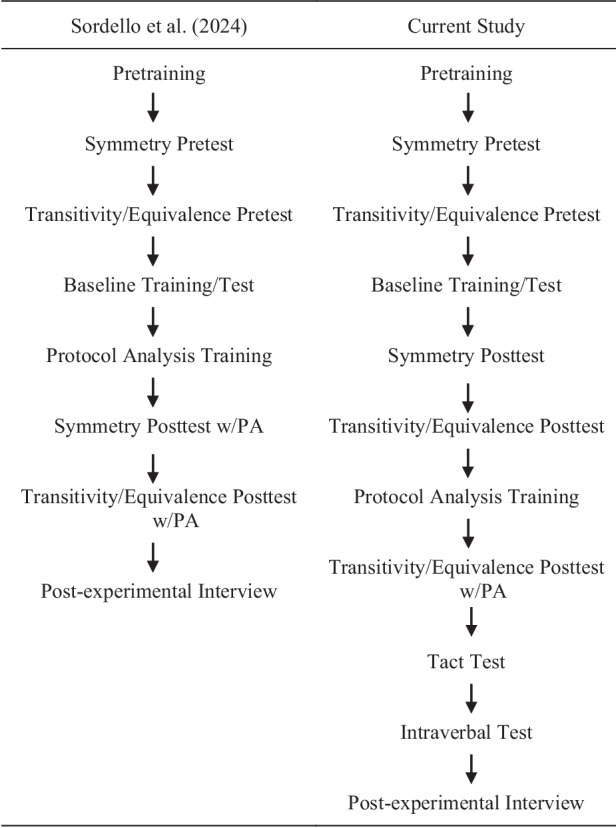
Procedural differences. *Note*. PA = protocol analysis

Conditions were presented in the following order: pretraining, symmetry (BA/CA) pretest, transitivity/equivalence (BC/CB) pretest, baseline training and test (AB/AC), symmetry posttest, transitivity/equivalence posttest, protocol-analysis training, transitivity/equivalence posttest with protocol analysis, tact test, and intraverbal test. Pretraining, symmetry and transitivity/equivalence pretests, and baseline training and testing were identical to Sordello et al. (2024; see Table 4 for summary of conditions). The current study differed from Sordello et al. by first requiring participants to complete transitivity/equivalence posttests before protocol-analysis training. Then, participants completed an additional block of the transitivity/equivalence posttest with the talk-aloud requirement. Moreover, participants completed tact and intraverbal tests following emergence posttests (see Fig. 1 for procedural differences).

**Table 4:** Summary of conditions

Condition	Summary
Pretraining	Familiarized participants with software. Participant were presented with familiar dictated words and required to respond correctly across eight consecutive trials to continue.
Symmetry pretest	Followed S-MTS trial presentation described in the “General Procedure” section. Participants were required to score 67% or below across one or two blocks. No prompts or reinforcement provided. Each block contained 24 trials and trials were randomized across blocks and participants.
Transitivity/equivalence pretest	Followed S-MTS trial presentation described in the “General Procedure” section. Participants were required to score 67% or below across one or two blocks. No prompts or reinforcement provided. Each block contained 36 trials and trials were randomized across blocks and participants.
Baseline training	Followed S-MTS trial presentation described in the “General Procedure” section. If participants did not touch related sample-comparison combinations, experimenter instructed participant to touch the white box at a 4 s delay. Correct go trials results in a tone and 10 points displayed at the top of the screen. No consequences or prompts were provided for correct no-go trials or incorrect trials. Participants were required to score 100% (without prompts) across two consecutive blocks to continue. Participants allotted 24 total blocks to reach criterion. Each block contained 24 trials and trials were randomized across blocks and participants.
Baseline test	Followed S-MTS trial presentation described in the “General Procedure” section. Participants were required to score 100% (without prompts or feedback from computer) across one block to continue. Participants returned to baseline training if they scored less than 100%. Each block contained 24 trials and trials were randomized across blocks and participants.
Symmetry posttest	Followed S-MTS trial presentation described in the “General Procedure” section. Participants were required to score 92% or above across two consecutive blocks for emergence criterion. Symmetry posttests terminated if stable or decreasing trend below emergence criterion was displayed across three consecutive blocks. Participants advanced to transitivity/equivalence posttests regardless of performance. No prompts or reinforcement provided. Each block contained 24 trials and trials were randomized across blocks and participants.
Transitivity/equivalence posttest	Followed S-MTS trial presentation described in the “General Procedure” section. Participants were required to score 94% or above across two consecutive blocks for emergence criterion. Posttests terminated if a stable or decreasing trend below emergence criterion was displayed across three consecutive blocks. No prompts or reinforcement were provided. Each block contained 36 trials and trials were randomized across blocks and participants.
Protocol analysis training	Participants chose a simple mathematical equation from a list of five and were instructed to talk out loud as to how they were solving the equation as if they were writing it on paper. Participants were required one correct response (by talking aloud rather than just stating the solution) to continue.
Transitivity/equivalence posttest w/protocol analysis	Followed S-MTS trial presentation described in the “General Procedure” section with protocol analysis. Participants were exposed to one block only. Vocal-verbal statements were coded as consistent, inconsistent, or irrelevant. Posttests were terminated if stable or decreasing trend below emergence criterion was displayed across three consecutive blocks. No prompts or reinforcement were provided. The block contained 36 trials and blocks were randomized across participants.
Tact test	Participants heard a sound from computer and had 5 s to respond. Responses were coded as experimenter-defined tacts or unique tacts. No prompts or reinforcement were provided. Participants were exposed to 18 trials with each stimulus presented twice and trials were randomized across participants.
Intraverbal test	Participants heard a sound from the computer and were instructed to respond with two sounds that went with the sound they heard and had 5 s to respond. Responses were coded as consistent, inconsistent, or irrelevant. No prompts or reinforcement were provided. Participants were exposed to 18 trials with each stimulus presented twice and trials were randomized across participants.

During training we utilized a one-to-many (OTM) format (i.e., participants were taught AB and AC relations; see Table 5). At trial onset, participants sat before a touchscreen computer in which the computer speakers played an auditory sample for approximately 3 s. After which a green box measuring 4.5 cm x 4.5 cm appeared at a 0-s delay. After touching it, the green box disappeared and an auditory comparison was played (0-s delay), followed by the appearance of a white box with a black outline measuring 4.5 cm x 4.5 cm, which remained on the screen for 8 s. Participants either touched (go) the box before the 8-s response interval elapsed or refrained from touching (no-go) the box for 8 s, depending on the relation between sample and comparison. When participants touched the white box, they were also instructed to say, “Click,” to provide an auditory indication to the experimenters that they were touching the box (Sordello et al., 2024). Touching related sample and comparison combinations during baseline training was followed by a tone from the computer speaker presented simultaneously with 10 points centered at the top of the monitor. No consequences or prompts were provided for responding correctly during no-go trials. Moreover, responding incorrectly during go and no-go trials produced no feedback. A 2 s inter-trial interval separated all trials and the white box remained in the window for a duration of 8 s.

**Table 5:** Related and unrelated combinations across S-MTS phases

Relations	Related (Go)	Unrelated (No-Go)	
AB/AC baseline relations	A1B1	A1B2	A2B3
	A1C1	A1C2	A2C3
	A2B2	A1B3	A3B1
	A2C2	A1C3	A3C1
	A3B3	A2B1	A3B2
	A3C3	A2C1	A3C2
BA/CA symmetry relations	B1A1	B2A1	B3A2
	C1A1	C2A1	C3A2
	B2A2	B3A1	B1A3
	C2A2	C3A1	C1A3
	B3A3	B1A2	B2A3
	C3A3	C1A2	C2A3
BC/CB transitivity/equivalence relations	B1C1	B1B2	B2B1
	C1B1	B1C2	C2B1
	B2C2	B1C3	B3B1
	C2B2	C1B2	C3B1
	B3C3	C1C2	B2C1
	C3B3	C1B3	C2C1
		C1C3	B3C1
		B2B3	B3B2
		B2C3	C3B2
		B3C2	C2C3
		C2B3	C3C2

*Note*. Baseline training and symmetry pre and posttests had 24 trials per block, whereas transitivity/equivalence pre and posttests had 36 trials per block. Twenty-four and 36 trial blocks were used because three, 3-member classes produce an unequal amount of related and unrelated stimuli combinations. To address this imbalance and to randomize the number of related (go) trials following unrelated (no-go) trials across conditions, related trials occurred twice, and unrelated trials occurred once per block

### Experimental Design

The study employed a nonconcurrent multiple baseline design across participant dyads (Watson & Workman, 1981). This design served to rule out the possibility that repeated exposure to stimuli would lead to stimulus-class formation prior to training. Participants in the first tier completed one block of symmetry and transitivity/equivalence pretests, whereas participants in the second tier completed two blocks of each pretest. Participants within the second tier were limited to two blocks to prevent the establishment of spurious stimulus relation(s), as well as fatigue.

#### Interobserver Agreement and Procedural Fidelity

Interobserver agreement (IOA) was assessed across all trials and conditions between the primary investigator and the computer program and across 95.5% of trials and conditions between the primary investigator and a secondary observer. Although the computer program recorded correct and incorrect go/no-go responses, a secondary observer was used in the event of a computer malfunction and to record responses during the protocol analysis, tact, and intraverbal tests. We calculated IOA by dividing the number of agreements by the sum of agreements and disagreements and multiplying by 100. Interobserver agreement averaged 100% between the primary investigatory and the computer, 99.7% (range, 99.4-100%) between the primary and secondary observers during S-MTS, tact, and intraverbal tests, and 99.3% (range, 97.2–100%) for statements during the protocol analysis.

Procedural fidelity (PF) was recorded by the secondary observer during all baseline-training trials for correct timing of the primary investigator’s prompt delivery during go trials (i.e., 4 s), and prompts only occurring during go trials. We calculated PF by dividing the number of correctly implemented baseline trials by the total number of baseline trials and multiplying by 100. Procedural fidelity averaged 99.8% (range, 95.8–100%).

## Results

**Fig. 2:**
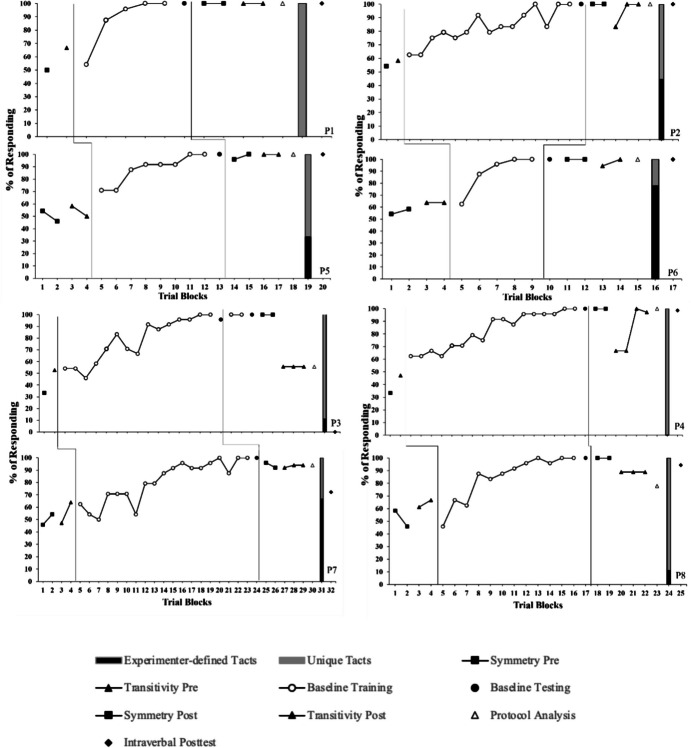
Percentage of correct go and no-go responses, percentage of unique and experimenter-defined tacts, and percentage correct during intraverbal test for P1–P8. *Note.* Transitivity/Equivalence w/PA = transitivity/equivalence posttest with protocol analysis (talk-aloud requirement)

Figure 2 depicts percentages of correct go/no-go responses across conditions. All participants scored 67% or lower on symmetry and transitivity/equivalence pretests and completed baseline training/testing following an average of 321 trials (range, 144–480). Emergence criterion for symmetry posttests was met within two blocks across all participants. Emergence criterion for transitivity/equivalence posttests was met by six participants (P1, P2, P4–P7) within two to four blocks. Average reaction times remained below 2 s (range, 0.66 s–5.15 s) across conditions, except for the posttest with the protocol analysis which averaged 2.19 s (range 0.9 s–5.8 s). Figure 2 also shows that most of the participants’ correct go and no-go responses were similar during transitivity/equivalence tests with and without the protocol analysis. The exception was P8 whose S-MTS performance deteriorated during the protocol analysis.

Figure 2 also shows the percentage of responses in the tact test that matched experimenter-defined tacts (black bars) and those that were unique (grey bars). During the tact test, six participants (P2, P3, P5–P8) engaged in a combination of experimenter-defined tacts (44.4%, 11.1%, 33.3%, 77.8%, 66.7%, & 11.1%, respectively) and unique tacts (55.6%, 88.9%, 66.7%, 22.2%, 33.3%, & 88.9%, respectively), and two participants (P1 and P4) engaged only in unique tacts. Six participants (P1, P2, P4, P5, P6, and P8) met emergence criterion for the intraverbal test.

**Fig. 3:**
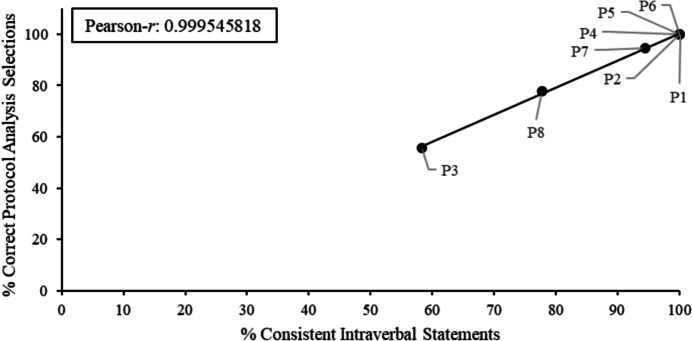
Percentage of consistent statements vs. percentage of correct selections for P1–P8

**Fig. 4:**
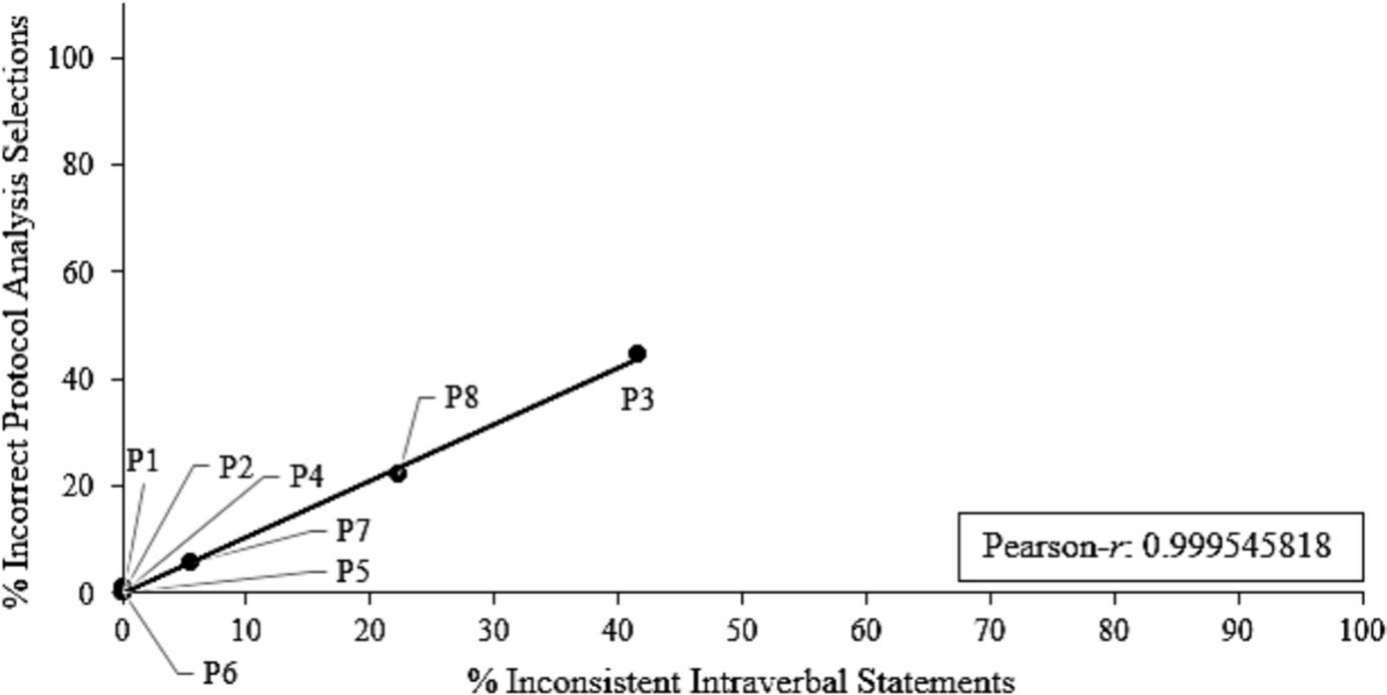
Percentage of inconsistent statements vs. percentage of incorrect selections for P1–P8

Figure 3 depicts correlations between the percentage of consistent intraverbal statements and percentage of correct selection responses during the transitivity/equivalence posttest with the protocol analysis, whereas Fig. 4 depicts correlations between inconsistent statements and percentage of incorrect selection responses during the transitivity/equivalence posttest with the protocol analysis. The experimenters calculated a Pearson-*r* and strong positive correlations between verbal behavior and S-MTS performances were found (r = 0.9995, for both correlations). Overall, participants (P1, P2, P4–P7) who engaged in class-consistent statements (i.e., over 90% class-consistent) during the protocol analysis emitted more correct S-MTS responses (i.e., over 90% correct selection responses), whereas participants (P3 and P8) who engaged in fewer class-consistent statements made fewer correct S-MTS responses. These are also the ones who did not meet the transitivity/equivalence emergence criterion. In general, participants emitted very few irrelevant statements during the protocol analysis, except for P8 who engaged in irrelevant statements more frequently (e.g., “I don’t know how they go together, they just go together,” etc.).

## Discussion

The purpose of the current study was to replicate Sordello et al. (2024) to evaluate the effectiveness of S-MTS in producing nonverbal auditory classes, assess whether a protocol analysis would influence transitivity/equivalence performance, and assess the possible role of verbal mediation during go/no-go S-MTS tasks. The results showed that six of eight participants formed equivalence classes. Moreover, the protocol analysis seemed to have impacted equivalence class formation, and there was evidence of possible verbal mediation during testing.

The fact that our equivalence yields (i.e., 75%) improved upon those of Sordello et al. (2024; i.e., 50%) and replicated studies that did not require a protocol analysis (e.g., Hanson & Miguel, 2021) suggests that requiring participants to talk aloud may, sometimes, interfere with their initial test performance. However, there are several studies that have employed a talk aloud requirement without any detrimental effects on testing (e.g., Cordeiro et al., 2021; Diaz et al., 2020; Meyer et al., 2019), albeit they all adopted a multiple-comparison MTS format. Therefore, it is important that future studies explore whether the talk-aloud requirement affects test performance and whether this is specific to the testing format.

Our study also attempted to assess the possible influence of participants’ verbal behavior on their S-MTS performance by including the talk aloud requirement (protocol analysis) during a final transitivity/equivalence posttest, as well as tact and intraverbal tests. Results showed that responses during tact and intraverbal tests were consistent with vocal-verbal statements made during the protocol analysis, despite previous studies having found that verbal behavior emitted during MTS tasks may differ from verbal behavior during independent probes (Meyer et al., 2019). Additionally, our results replicated those of Sordello et al. (2024) in that consistent statements and correct selections, and inconsistent statements and incorrect selections co-varied. These results add additional evidence to the notion that supplemental discriminative stimulation arising from participants’ verbal behavior may play a role in emergent performance (e.g., Chastain et al., 2022; Jennings & Miguel, 2017; Lee et al., 2015).

A few limitations are worth mentioning. First, this study was conducted in person, whereas Sordello et al. (2024) was conducted remotely, which may prevent comparisons. However, Hanson and Miguel (2021) found no statistically significant differences in equivalence yields when S-MTS was conducted remotely as compared to in-person. Second, although participants were required to touch the green box after the presentation of the auditory sample to activate the comparison, it is unclear whether participants were always attending to the sample stimulus. Thus, future research should consider adding a trial-initiation and/or a differential observing response for both samples and comparisons.

In conclusion, the current study adds to the evidence, at least with adult participants, for using go/no-go S-MTS to establish equivalence classes with nonverbal stimuli and to produce tacts and intraverbals. Despite its correlational nature, our results also add support to the notion that equivalence-class performances may be influenced by participants’ self-generated verbal behavior (Miguel, 2016, 2018). Finally, along with Sordello et al. (2024), we showed preliminary evidence that requiring participants to talk aloud (i.e., protocol analysis) during initial equivalence testing could negatively impact their performance. Thus, future research should continue to examine this, possibly conducting group designs with and without a protocol analysis during posttests.

Although there is plenty of evidence for the effectiveness of S-MTS in establishing conditional relations with college students in laboratory preparations across a variety of stimulus modalities, no applied studies have been conducted. Thus, future research should investigate the utility of S-MTS for teaching listener behavior and categorization to children with and without disabilities, as this procedure may be beneficial for those who may have difficulties learning simultaneous discriminations.

## Data Availability

All data are available upon request.

## Footnotes

1 When auditory stimuli were non-verbal such as the sound of a rocket launching, some participants engaged in unique tacts such as saying, “explosion.”
